# Differences in the ratio of soil microbial biomass carbon (MBC) and soil organic carbon (SOC) at various altitudes of Hyperalic Alisol in the Amazon region of Ecuador

**DOI:** 10.12688/f1000research.22922.1

**Published:** 2020-05-26

**Authors:** Benito Mendoza, Jaime Béjar, Daniel Luna, Miguel Osorio, Mauro Jimenez, Jesus R. Melendez

**Affiliations:** 1 Universidad Nacional de Chimborazo, Riobamba, Ecuador; 2 Escuela Superior Politécnica de Chimborazo, Riobamba, Ecuador; 3 Facultad Educación Técnica para el Desarrollo, Universidad Católica de Santiago de Guayaquil, Guayaquil, Ecuador; 4 Dama Research Center limited, Kowloon, Hong Kong

**Keywords:** microbial biomass carbon (MBC), soil organic carbon (SOC), MBC/SOC ratio, rainforest, Hyperalic Alisol, and Amazon region Ecuador

## Abstract

Protecting soil fertility represents a fundamental effort of sustainable development. In this study we investigate how different altitudes affect soil microbial biomass carbon (MBC) and soil organic carbon (SOC), and their ratio, MBC/SOC in Hyperalic Alisol. MBC and SOC are well established and widely accepted microbial quotients in soil science. Our work hypothesis was that a decrease in MBC and SOC should be observed at higher altitudes. This initial assumption has been verified by our measurements, being attributed to the increase in MBC and SOC at low altitudes. Our approach should contribute to the better understanding of MBC and SOC distribution in soil and changes in MBC/SOC at various altitudes in the region.

## Introduction

Protection of soil quality is a pillar of sustainable development ^1– 3^. Recent studies considered effects of minerals ^4– 6^ and soil cultivation techniques ^7, 8^ on soil quality and soil microbial activity ^9– 12^. These studies reported interactions between soil minerals and decomposition of organic matter; positive correlations between soil microbial activity (measured by number of bacteria and fungi, soil respiration, and C and N cycle-related enzyme activity) and cultivation methods, and fertilizer usage. The undertaken efforts in agriculture practice, offer favorable conditions for soil-plant interactions. Additionally, these studies on soil respiration and enzymatic activity report correlation analyses between soil properties and microbial activity, documenting that soil quality is mainly determined by soil microbial activity, as verified by other authors’ work ^13– 15^. A highly cited review paper on the topic ^16^ concludes that soil processes and properties are affected by climate change, which causes changes in soil organic matter (SOM) and microbial biomass carbon (MBC). Based on this, one can state that monitoring the soil carbon cycle is of increasing importance, given that microbial processes in soil may serve as an indicative of climate change. Among processes to be monitored, decomposition of organic matter is of primary significance ^17^, considering that this process is strongly influenced by soil microbiological activity. Soil management is another research area of increasing interest ^18^ owing to its significance in soil microbial activity ^19^ that can be expressed by enzymatic activity, soil respiration, and MBC ^11^. Local farmers have demonstrated willingness to start permaculture systems involving tropical orchards, like those previously operated successfully in another plant, Guayusa ( *Ilex guayusa*) ^20^, in the Amazon region. Because of the sensitivity of orchards to nutrient deficiency ^21– 23^ we believe that measuring soil microbial activity is of priority when analyzing soil properties, as soil microbial activity is in strong correlation with nutrient uptake in plants ^24^. For this reason, monitoring changes of soil MBC and soil organic carbon (SOC) allow to select favorable locations for orchard plantation in permaculture systems, contributing to sustainable agricultural practices. MBC is the living microbial component of soil organic matter ^25^ and is considered an indicator of microbial activity, owing to its rapid response (less than a year turnover time) to conditions that may alter soil organic matter ^26^. SOC contributes positively to soil fertility and crop production ^27^. Here we report variations with altitude of MBC, SOC, and MBC/SOC in Hyperalic Alisol.

## Methods

### Soil sampling

We were inspired by a prior study conducted in 2018 ^28^, in which soil properties at various altitudes were measured in the region. We collected samples at similar altitudes as in the prior study ^28^ (395, 1006, and 1554 m.a.s.l, meters above sea level) to gather data on soil properties at different altitudes. We collected 15 soil samples, 5 from each altitude, following the protocol proposed by Singla and co-workers ^28^, where a minimum of 3 sampling points per altitude were recommended: 420, 1000, and 1600 m.a.s.l. in the Ecuadorian Amazonian region (latitude: 4.628247894396525; longitude: -74.95615214109422), on December 10, 2019. Homogeneous soil populations can be used for analysis, when sample size (s) = 15, as determined by Cline (1944) ^29^ ( Equation 1):

$$s=\frac{r_{n}-r_{1}}{C}\;(E1)$$

Where,

r _n_, r _1_ are the extremes of the range of a parameter,

C is a constant, equal to 3, 4, 5, and 6 for 10, 25, 100, and 500 sampling units, respectively (Cine, 1944) ^29^.

To estimate extremes of a given range, we used prior literature data ^24^ rom a study performed in the Amazon region in which SOC values vary between 34.69 and 54.62. Cline (1944) ^29^ recommended the use of 20 samples, when the range of parameter is 110. In our case, counting with 19.93 range value obtained from a prior study in the region ^24^ Equation (1) becomes s = 19.93/4 = 4.98 ~ 5 samples. So, the total number of samples for the study is Total number of Samples = 5 samples multiplied with the three altitudes, yielding a number of 15 sampling points.

Samples originated from the upper layer (top 20 cm) of Hyperalic Alisol (Ultisols in U.S. Soil Taxonomy) soil. Sampling points and physical-chemical soil properties were described in a prior study ^30^, parameters being in the following ranges: pH 4.99–5.98; moisture content: 43.9–66.6 %; Allophane: volcanic; and total Fe content (mg/kg): 367–1104.

### MBC/SOC

MBC was measured in quadruplicate (see supporting data) by the fumigation-incubation method, according to Jenkinson and Powlson ^31^. Briefly, four aliquots of oven-dried (105 °C for 24 h) soil samples (25 g each) were placed into glass vials, two aliquots being fumigated with CHCl _3_ (Fisher Scientific, 67-66-3,64-17-5) at room temperature, while two aliquots were kept untreated at 2 °C for 24 h. All samples were incubated at 25 °C for 10 days, in the dark. Their respiration was measured after incubation with Barcroft-type (differential) respirometer, which enables simultaneous measurements of gas volume in real-time ^32^. Produced raw CO _2_ values (SIR) were read by Respirometer software RV10 (version 10.03) obtaining data directly from the respirometer’s electronic sensor (model: Respicond VIII, Nordgren Innovations, Sweden). Soil respiration of both fumigated and unfumigated samples were calculated from SIR values according to Anderson and Anderson (1978) ^32^ using the equation ( Equation 2):

Fumigated,Unfumigated=(40.4*SIR)+0.37(E2)

MBC was calculated by dividing the difference in soil respiration between fumigated and unfumigated samples with 0.38 according to Jenkinson and Powlson ^31^ ( Equation 3):

$$MBC=\frac{Fumigated-Unfumigated}{0.38}\;(E3)$$

Where,

Fumigated is the produced CO _2_ by fumigated soil samples, expressed in C µg/ml.

Unfumigated is the produced CO _2_ by not fumigated soil samples, expressed in C µg/ml.

To calculate SOC we determined humus content according to Székely *et al*. ^33^. In this procedure, 1.0 g of air-dried soil was placed into a 300-mL Erlenmeyer flask. Then, 10. mL of 5% K _2_Cr _2_O _7_ (Fisher Scientific, 7778-50-9) solution was added and mixed with the soil. Next, 20 mL of concentrated H _2_SO _4_ (Fisher Scientific, 7664-93-9) were supplemented, and mixed with 100. mL distilled water. Solution was filtered with Grade 42 Whatman slow filter paper (pore size 2.5 μm) and analyzed with Model 240Z Atomic Absorption Furnace Spectrophotometer (Agilent), from 230 to 700 nm, and absorbances at 600 and 400 nm ( *E _600_* and *E _400_*), respectively, correspond to A,B, P, and Rp types of humic acids, relevant to humus content, according to Watanabe *et al*. ^34^. Humus content varied from 2.34 (at 1600 m.a.s.l.), to 4.82, at lower laying areas (420 m.a.s.l.) (see raw data in Supporting material.) From the obtained humus content, we calculated SOC, using a conversion factor, according to Walkley and Black ^35^ ( E3):

SOC=humus(percent)/1.32(E3)

MBC/SOC quotient was determined by dividing MBC with SOC.

### Statistical analysis

Linear regression (Z-test) was performed to reveal possible statistical differences (p<0,05) between parameters and altitudes, using SPSS (version 26).

## Results and discussion

Our results are similar in magnitude with prior studies in the Amazonian region ^36^; the lowest SOC and MBC values are observed at the highest altitude (1600 m.a.s.l.): 1.77% and 267.3 mg/kg, where the average MBC/SOC is 161.17. At 1000 m.a.s.l. both SOC and MBC increased; SOC augmented by 74%, while MBC by 45%. Average MBC/SOC increased to 268.2. At the lowest altitude (420 m.a.s.l.) both SOC and MBC reached their highest values: 3.65% and 2214.4 mg/kg, respectively. The average MBC/SOC also reached its maximum: 667.8. Altitude significantly affects both SOC (R ^2^ = 0.95) with p< 0.0001 and MBC (R ^2^ = 0.89) with p< 0.0001 ( Table 1 ^37^).

**Table 1.** Correlation coefficients (R ^2^) between soil properties and altitudes. * represents significant correlation at 95% probability as accepted in agriculture ^31^. Note: humus % is not included in the table, given that SOC is calculated from humus %, as indicated in the methods section.

Variables	Altitude	SOC	MBC	MBC/SOC
Altitude	1	0.95*	0.89	0.88
SOC	0.95*	1	0.85	0.8
MBC	0.89	0.85	1	0.98*
MBC/SOC	0.88	0.8	0.98*	1

SOC - soil organic carbon, MBC - microbial biomass carbon

**Figure 1.**
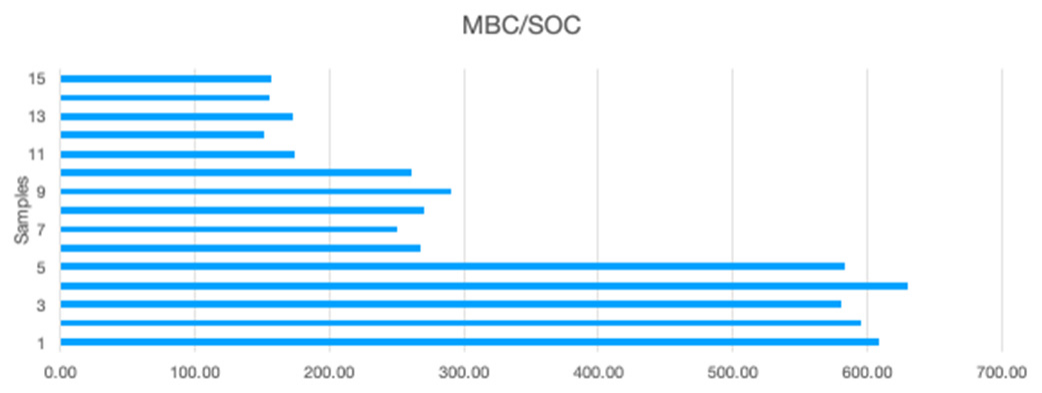
Changes in microbial biomass carbon (MBC)/soil organic carbon (SOC) at different altitudes. Samples numbered correspond to the following altitudes 1–5: 420 m.a.s.l.; 6–10: 1000 m.a.s.l.; 11–15: 1600 m.a.s.l.

MBC/SOC increases with decreasing altitude ( Figure 1 ^37^), which means that MBC increases more rapidly with decreasing altitude than SOC. As MBC corresponds to microorganisms’ weight (mainly bacteria and fungi), and is estimated at approx. 5% of the SOC ^32^, the increased MBC value indicates faster microbial activity at lower laying sampling points. This finding yields three main observations:

1. Organic matter decomposition is accelerated by soil microbial activity ^38, 39^ in lower-laying areas, because of more favorable conditions ^40^ offered to cellulose decomposing bacteria. Presence of these bacteria is confirmed by MBC/SOC increase at lower altitudes, reported here. Microbial carbon increases more significantly than organic carbon, which suggests increased microbial activity ^40^.
2. Availability of SOC depends on soil properties, and soil depth ^41^, rather than on altitude or coverage ^42^. SOC may also depend on several other soil properties, therefore, enzymatic activities and physical-chemical soil properties should be measured. Total iron increases with decreasing altitude, as well ^30^. With these findings in MBC/SOC value changes, general leaching can be documented.
3. Metabolic efficiency depends on the availability of substrate (organic matter). The greater the MBC, the greater the temporary immobilization of micro and macro-nutrients ^43– 46^. To confirm this, immobilization of nutrients should be measured in soil-plant interactions (mainly uptake), as planned for future work in which orchards will be investigated, given the local farmers’ need to plantation in the area and the particular sensitivity of orchards to nutrient deficiency ^21– 23^.

## Conclusions

Altitude affects SOC significantly. Decrease in MBC/SOC quotient is observed with increasing altitude. From obtained results we can conclude that lower-laying areas favor increased soil microbial activity. We recommend lower-laying areas for orchard plantations, considering that orchards are particularly sensitive to nutrient deficiency ^47^, while soil microbial activity is in strong correlation with nutrient uptake in plants ^24^.

## Data availability

### Underlying data

Figshare: Supporting raw data for MBC and SOC. Dataset. https://doi.org/10.6084/m9.figshare.12264698.v1 ^37^.

This project contains the following underlying data:

- Supporting Data_ Melendez-V3.xlsx (raw respiration data for MBC calculation and ABS raw data for humus % and SOC calculation)

Data are available under the terms of the Creative Commons Attribution 4.0 International license (CC-BY 4.0).
