# Supplementary material for: Differences in the ratio of soil microbial biomass carbon (MBC) and soil organic carbon (SOC) at various altitudes of Hyperalic Alisol in the Amazon region of Ecuador
Source: F1000Res. 2020 May 26;9:443. [Version 1] doi: 10.12688/f1000research.22922.1 (PMC7281642; doi:10.12688/f1000research.22922.1)
Supplement: Peer review history

# Peer review: Reviewer response for version 1

**DOI:** 10.5256/f1000research.25306.r63948

**Published:** 2020-06-08

**Authors:** Istvan Fekete

**Affiliations:** 1 Institute of Environmental Sciences, University of Nyíregyháza, Nyíregyháza, Hungary

A very accurate latitude and altitude data are given for the location of the site, which belongs to one specific site; however, there are 3 different altitudes in the text, at which 3-3 sampling points per altitude were mentioned.

 In the Material and Method, it would have been good to include a site description section, in which the vegetation of the study area could have been described. Also describing what is grown in agricultural areas; how much biomass remains in the area; how long the natural vegetation has been removed (since when the area has been under cultivation). It would be good to know if there was a difference between the slope of each slope because this can also affect SOM and MBC through erosion.

 Soil moisture and temperature fundamentally affect the MBC of soils, so it would be good to mention the values for specific sites in this manuscript as well. Furthermore, it would be good to know the particle composition of the soil, as the rate of the clay fraction significantly influences the SOC content.

 The above-mentioned variables and parameters can all influence the final result.

Is the work clearly and accurately presented and does it cite the current literature?

Yes

If applicable, is the statistical analysis and its interpretation appropriate?

Yes

Are all the source data underlying the results available to ensure full reproducibility?

Partly

Is the study design appropriate and is the work technically sound?

Partly

Are the conclusions drawn adequately supported by the results?

Yes

Are sufficient details of methods and analysis provided to allow replication by others?

Partly

Reviewer Expertise:

Soil ecology, soil carbon content

I confirm that I have read this submission and believe that I have an appropriate level of expertise to confirm that it is of an acceptable scientific standard.

# Peer review: Reviewer response for version 1

**DOI:** 10.5256/f1000research.25306.r63917

**Published:** 2020-06-01

**Authors:** Lenin Ramirez-Cando

**Affiliations:** 1 Grupo de Investigación Ambiental en el Desarrollo Sustentable GIADES, Carrera de Ingeniería Ambiental, Universidad Politécnica Salesiana, Quito, Ecuador

The authors report changes in soil microbiological parameters and their ratio at different altitudinal levels of the Ecuadorian Amazon region. The study is well written and straightforward; anyways, in parts needed to be improved to increase readability, and lead readers to better understand the importance of the research findings.

 First, in the introduction, authors discuss the role of MBC and, then SOC in assessing soil microbiological activity, but what missing for me is the lack of explaining their ratio MBC/SOC as a microbial indicator. The importance of ratio is mentioned in the paper, but its role is unclear. 

 Up to date and relevant literature is cited. Methods are well written, but missing some information. How many samples were measured for MBC and SOC, 15 (as sampled) in quadruplicated? It is not clear until we open the supporting material. 

 Observation: generally in mobilization studies, apart from the altitudinal variant, sampling depths are also involved in the analysis. It is not clear to me why the authors disregarded different depths in the study that could provide a more complex image of examined changes. 

 Results are presented logically, and succinctly. Conclusions are correct. Overall, it can be said that it is a well-designed study. In any case, I suggest making the following minor changes before article indexing: 

- Expand introduction, and highlight the role of ratio MBC/SOC.
- State number of samples used for biomass analyses.
- Please clarify why depth was not involved in the analysis.

Is the work clearly and accurately presented and does it cite the current literature?

Yes

If applicable, is the statistical analysis and its interpretation appropriate?

Yes

Are all the source data underlying the results available to ensure full reproducibility?

Yes

Is the study design appropriate and is the work technically sound?

Yes

Are sufficient details of methods and analysis provided to allow replication by others?

Partly

Reviewer Expertise:

NA

I confirm that I have read this submission and believe that I have an appropriate level of expertise to confirm that it is of an acceptable scientific standard.
